# How health professionals perceive and experience treating people on social assistance: a qualitative study among dentists in Montreal, Canada

**DOI:** 10.1186/1472-6963-13-464

**Published:** 2013-11-05

**Authors:** Christophe Bedos, Christine Loignon, Anne Landry, Paul J Allison, Lucie Richard

**Affiliations:** 1 Division of Oral Health and society, Faculty of Dentistry, McGill University, 3550, rue University, Montréal, Québec H3A 2A7, Canada; 2 Institut de recherche en santé publique de l’Université de Montréal (IRSPUM), Montréal, Quebec, Canada; 3 Faculty of Medicine, University of Sherbrooke, 150 Place Charles Lemoyne Bureau 200, Longueuil, Québec J4K 0A8, Canada; 4 Agence de la Santé et des Services Sociaux de Montréal, 1301, rue Sherbrooke Est, Montréal, Québec H2L 1M3, Canada; 5 Faculté des Sciences Infirmières, C.P. 6128, Succursale Centre-ville, Montréal, Québec H3C 3J7, Canada

**Keywords:** Poverty, Social assistance, Health Care, Dentists, Qualitative research, Semi-structured interviews

## Abstract

**Background:**

In Canada, the prevalence of oral diseases is very high among people on social assistance. Despite great need for dental treatment, many are reluctant to consult dental professionals, arguing that dentists do not welcome or value poor patients. The objective of this research was thus to better understand how dentists perceived and experienced treating people on social assistance.

**Methods:**

This descriptive qualitative research was based on in-depth semi-structured interviews with 33 dentists practicing in Montreal, Canada. Generally organized in dentists’ offices, the interviews lasted 60 to 120 minutes; they were digitally recorded and later transcribed verbatim. The interview transcripts were coded with NVivo software, and data was displayed in analytic matrices. Three members of the research team interpreted the data displayed and wrote the results of this study.

**Results:**

Dentists express high levels of frustration with people on social assistance as a consequence of negative experiences that fall into 3 categories: 1) Organizational issues (people on social assistance ostensibly make the organization of appointments and scheduling difficult); 2) Biomedical issues (dentists feel unable to provide them with adequate treatment and fail to improve their oral health); 3) Financial issues (they are not lucrative patients). To explain their stance, dentists blame people on social assistance for neglecting themselves, and the health care system for not providing adequate coverage and fees. Despite dentists’ willingness to treat all members of society, an accumulation of frustration leads to feelings of powerlessness and discouragement.

**Conclusions:**

The current situation is unacceptable; we urge public health planners and governmental health agencies to ally themselves with the dental profession in order to implement concrete solutions.

## Background

The burden of oral diseases is high among underprivileged people in North America and constitutes a serious public health issue [1,2]. Yet, despite a high prevalence of oral diseases and a great need for dental treatment, most people living in poverty rarely consult a dentist [3-5]. This applies particularly to people on social assistance, who could benefit from public dental insurance programs. In the United States, for instance, use of dental care services is very low among Medicaid recipients, whether adults or children [6,7]. A similar phenomenon occurs in Canada: in the province of Quebec, people on social assistance often adopt a wait-and-see approach and try to adapt to symptoms rather than consult an oral health professional [8-10]. In brief, public dental insurance programs for people living in poverty are insufficient to ensure timely use of dental services and eliminate disparities in access.

Several factors could explain this disturbing situation: the dental care pathway for people on social assistance depends on how they define oral health [11], oral illness and need for treatment [12]; it is also impeded by indirect costs related to transportation [13] and services not covered by public insurance [10]. In addition, the relationship with oral health professionals seems to be a crucial element. Indeed, many people on social assistance report negative experiences with the dental care system: in the United States, for instance, Medicaid enrolees have described disrespectful and discriminatory attitudes by oral health professionals toward them because of their status [14]. In Canada as well, people on social assistance have reported complicated relationships with dentists and deplored a lack of sensitivity to their situation [10].

For their part, many dentists in North America seem reluctant to treat people on social assistance. Indeed, in the United States, dentists’ participation rate in the Medicaid program remains low [6] for various reasons: cancelled appointments, low reimbursement rates, poor compliance, and complicated paperwork [15-17]. Despite these findings, we actually know very little about how dentists experience treating people on social assistance. One of the reasons is that previous studies relied on structured questionnaires; based on deductive approaches, they did not favor the emergence of new perspectives and tended to provide de-contextualized data. We therefore decided to conduct qualitative research whose objective was to better understand how dentists perceived and experienced treating people on social assistance. In particular, we were interested in deepening our understanding of the difficulties that they may encounter with this group of people.

## Methods

### Research design

We used a descriptive qualitative research design based on open-ended, semi-structured interviews. Qualitative methodologies are indeed indicated for exploring complex phenomena about which little is known [18]. The flexibility of the design, consisting of simultaneous data collection and analysis, also allowed us to sample on the basis of emerging concepts and gather information on topics that derived from the first analyses.

### Sampling strategy

We conducted this study in Montreal, Canada, a multicultural city with 1.6 million inhabitants [19] and almost 1400 general dental practitioners [20]. We adopted a maximum variation sampling strategy [21] to recruit general dentists with potentially diverse experiences with people living in poverty. In particular, we wanted to meet clinicians with various years of experience, working in several types of settings (multi practice, solo practice) and with different professional status (owners, employed). This is why, for instance, we contacted dentists practicing in diverse types of neighbourhoods, including underprivileged areas and affluent districts.

We recruited the dentists by sending a written invitation (by mail, email, or fax) and a subsequent telephone call to plan an interview. In the written invitation as well as during the phone call, we informed them that they had no obligation to participate and could take the time they needed to decide on whether to participate or not. We stopped recruiting when we obtained data saturation, “the point at which additional data does not improve understanding of the phenomenon under study” [18] and simply reiterates what has been previously collected.

### Data collection

Experienced interviewers collected data between 2004 and 2007 through in-depth, semi-structured interviews. Generally organized in participating dentists’ offices, the interviews lasted 60 to 120 minutes; they were digitally recorded and later transcribed. Before each interview, the participants were invited to read a consent form approved by the academic ethics committee of McGill University’s Faculty of Medicine. We encouraged them to ask questions about the research and their rights as participants prior to signing the consent form.

Researchers used an interview guide that focused on dentists’ experiences with people on social assistance. This guide was designed to help interviewers identify the problems and difficulties faced by dentists. In order to obtain more in-depth information on the topics discussed, researchers used “probing” techniques [21]: when necessary during the course of the discussions, they formulated follow-up questions that also allowed them to explore unanticipated but relevant emerging topics.

### Data analysis

We performed a thematic analysis, a “method for identifying, analysing and reporting patterns (themes) within data” [22]. The analysis comprised several parts: interview debriefing, transcript coding, data display and interpretation.

The debriefings were conducted between the interviewer and the main researcher after each interview. They served to summarize the main findings, identify emerging hypotheses, and prepare subsequent interviews. Coding of the interview transcripts was carried out using NVivo software. We used an initial list of codes inspired by the research questions, but refined this list throughout the coding. The process involved cutting the transcripts into meaningful segments and assigning codes to the segments. We then examined the codes and their corresponding passages through an iterative process, grouping them into broad themes and displaying them in analytic matrices, as recommended by Miles [23]. We finally described the themes in a text and illustrated them with excerpts of the data.

To improve the rigor and credibility of our results, three members of the research team conducted this process, checking and validating their analysis. In particular, they coded initial transcripts separately and then compared their findings; for each instance of coding disagreement they discussed their interpretations, refined the codes, and undertook coding again until agreement was reached. Furthermore, the researchers carefully pondered the analytic matrices while comparing their interpretation of the results. Again, when confronted with divergence, they discussed the data until jointly able to agree upon an interpretation.

### Description of the sample

A total of 33 dentists, 21 men and 12 women, participated in the study (Table 1). They ranged in age from 26 to 70 years and had practiced dentistry for periods of between 2 and 45 years. Fifteen participants identified themselves as having a non-western ethno-cultural background, which included Iranian, Lebanese, Armenian and Vietnamese origins.

**Table 1:** Sample description (N = 33)

**Categories**	**N**
Age (years)	
21-30	6
31-40	8
41-50	9
51-60	5
61 or more	5
Gender	
Female	12
Male	21
Background	
Western background (Canadian)	18
Non-western background (Non-Canadian)	15
Years of experience as a dentist	
0-5	2
6-15	14
16-30	9
31 or more	8
Type of clinical setting	
Multi practice	21
Solo practice	12
Professional status	
Owner (or co-owner)	25
Employed (paid by percentage)	8

## Results

When asked about their relationship with people on social assistance at the dental office, most participating dentists express high levels of frustration and sometimes anger. Frustrations were the consequence of negative experiences that fell into 3 broad categories: 1) Organizational issues; 2) Biomedical issues; 3) Financial issues (Tables 2 and 3). Despite these dentists’ efforts and willingness to treat all members of society, the accumulation of frustration led to feelings of powerlessness and discouragement.

**Table 2:** Issues identified by participating dentists

**Types of issue**	**Problems reported by dentists**	**Explanations provided by dentists**	**Main sources of the issues according to dentists**
**Organizational**	Dentists find the organization of appointments and scheduling difficult for people on social assistance	● People on social assistance tend to consult in an emergency	● People on social assistance
		● They often miss appointments and have no valid reasons for that	● People on social assistance
		● They have little availability during off-peak hours	● People on social assistance
**Biomedical**	Dentists feel unable to provide them with adequate treatments and improve their oral health	● Public dental insurance does not cover several treatments	● Public health care system
		● People on social assistance lack motivation to care for their health	● People on social assistance
**Financial**	Dentists perceive patients on social assistance as non lucrative and a threat to the financial sustainability of their clinic	● The reimbursement rates of the government are too low	● Public health care system
		● The treatments performed are often basic	● People on social assistance
		● Missed appointments cause lost wages	● People on social assistance

**Table 3:** Illustrating quotes

**Themes**	**Quotes**
**Organizational Issues** – **Dentists find the organization of appointments difficult for people on social assistance**	
People on social assistance tend to consult in an emergency and often miss appointments	*That, I’ll be honest, is one of the things that bugs me the most even now. They don’t show up. They don’t show up, they don’t call, they don’t let us know, and that’s just how it is.* [CL3]
People on social assistance do not have valid reasons for missing appointments	*“I went to bed at 4 in the morning. I didn’t feel like coming.” They’ll tell you. They went to a bar, they went out to a strip show. They were watching t.v. They went out on the town with their buddies. And then, well, of course, when you go to bed at 4 and you have an appointment at 10 in the morning, well, sometimes…* [CL12]
People on social assistance are not flexible and have little availability during *off-peak hours*	*It’s simple; you don’t give a morning appointment to someone on social assistance because that’s when he sleeps! […] They tell us: “not in the morning, not too early in the morning. Not 9 a.m. Not 10 a.m. We get up late.” Fine. “So we’ll give you one in the afternoon.” “Well, no; I work.” […] So these patients want to come in the evening when I don’t work, so they’ll come on a Saturday and take the spots of my good patients’, who make an honest and steady living.* [CL13]
**Biomedical issues - Dentists feel unable to provide people on social assistance with adequate treatments**	
Public dental insurance does not cover several treatments; people on social assistance cannot afford to pay for treatments not covered	*It is unsatisfying to be very limited in the treatments I can offer people on assistance. The treatments we can offer people on assistance are treatments that date back to the thirties, to the fifties. […] So for someone who likes technology a lot, who likes to perform a lot of state-of-the-art treatments, it’s disappointing. I have to say that, usually, I get no pleasure from treating people on social assistance; it’s not because they’re on social assistance, it’s because of the limits placed on me in terms of treatment options. [AL10]*
People on social assistance lack motivation to care for their health and neglect themselves	*I have experienced the same thing my colleagues have; we all have. Patients on welfare are not always reliable.* [CB2]
Dentists feels powerless and discouraged	*We try telling them, « floss and brush your teeth ». There is often a generalized level of neglect. The [dental] hygienist, for example, often gets really discouraged.* [AL5]
**Financial issues – Dentists perceive patients on social assistance as non lucrative and a threat to financial sustainability**	
The government fee schedule is too low	*Even though we’re paid, when it comes down to it, it’s almost pro bono work. Because to, say, remove a tooth, I think it’s something like 13 dollars. It costs me more in electricity, material, my assistant, my secretary, and all that, than what I make.* [AL3]
The low reimbursement rates are unfair and frustrating	*It annoys me to be paid less by* [public] *health insurance. It really annoys me because I don’t take less good care of that particular patient. I can’t sterilize the instruments less. I can’t use poorer quality materials. I only have one kind of amalgam, so I use it for everyone. Of course, maybe, for another [person not on public assistance], I might give them a crown, but you can’t work less well just because someone is on social assistance. So I don’t know why we shouldn’t be paid the same price. But instead of giving to the United Way [charity], I give to the public insurance scheme.* [CL8]
People on social assistance's missed appointments create a “wage gap”	*Given that, first of all, just seeing them pays less than the same procedure I would give to you or someone who has* [private] *insurance, I don’t get the same fees. And if I’ve scheduled an hour for restorations and he doesn’t show up, plus, you know, in addition to losing the fees I would have had with a normal patient, I lose the hour as well.* [CL2]
People on social assistance are a threat to financial sustainability	*Currently, I have very few. I don’t mind at all. It’s not like I have a big clientele of only them…* [CB2]
	*Dentist: I like my work. Except for the fact that my colleagues make more money than I do, and they’re always rubbing my face in it, and I have to live with it.* […] *The only thing… society judges success according to how much money we make, so psychologically it has an effect [on me], to see that I am below average, I guess, compared to other dentists.* [AL4]

### Organizational issues – Dentists find the organization of appointments and scheduling difficult for people on social assistance

Dentists complain that people on social assistance generate problems with respect to the organization of appointments: not only do these patients tend to consult in an emergency but, when appointments are scheduled, they often reportedly fail to show up. Dentists add that problems persist even after the “phone recall” that the dental office’s secretary makes to confirm a patients’ presence the day before the appointment. Dentists deplore the resulting disorganization of their schedule, causing them to lose time, and preventing other patients from being promptly treated.

*The child supposedly is in pain because he has a big cavity. We’ll say: “Ok, fine, we’ll schedule him as soon as possible”. And then, [it’s] a missed appointment. You know, it’s frustrating because the kid was in pain, it’s free, he has an appointment, we want to treat him, we make room and then, missed appointment. So in the long run, you develop a kind of [attitude]: “Oh, no; not a welfare patient!”* [CL2].

Furthermore, dentists argue that people on social assistance rarely call to cancel. This irritates them because they consider “no-shows” to signal a lack of respect towards them. For many, the frustration is reinforced by their perception that people on social assistance do not have serious reasons for missing their appointments, considering the fact that they benefit from public dental insurance: “no-shows” purportedly reflect a lack of motivation and laziness on the part of people on social assistance.

*It’s because they don’t wake up in time, it’s not nice out, it’s too nice out, they have the flu. Excuses. Except that at a certain point, when you schedule five appointments and he misses four of them, with different excuses… my tolerance decreases after the third one.* [AL5].

Another frustration that dentists express relates to difficulty planning this clientele’s appointments outside of peak periods. Considering that the latter do not have a job, dentists expect them to be flexible and thus available when the dental office is less crowded, such as in the morning or early afternoon. However, people on social assistance often refuse to book appointments at those times for reasons that dentists attribute to their lifestyle: for instance, they pinpoint their laziness or, on the contrary, undeclared jobs that impede scheduling. As a result, some dentists resent treating people on social assistance during time slots intended for “good patients”.

*Even though they didn’t work, they weren’t available as we would have wanted, like in the morning. They could demand an evening appointment. The secretary was a bit bothered by that. […] She of course complained: “So, they don’t work; why don’t they come in the morning?” Because morning appointments are always much harder to book.* [CB1].

### Biomedical issues – Dentists often feel unable to provide people on social assistance with adequate treatment or to improve their oral health

The dentists regret their general inability to provide people on social assistance with the best treatments available, which is due to two factors: the limitations of the public dental insurance program and a lack of motivation by people on social assistance toward improving oral health. With respect to the public dental insurance program, the dentists consider that, although most basic treatments are covered, several important procedures are not, such as root canal therapies (endodontic treatments). This case is particularly frustrating to them because patients on social assistance are generally unable to pay the fees and instead opt for extractions, a procedure that is fully covered by the government.

*Even if I offer to let them pay bit by bit, it doesn’t work at all. [The patients say] “No, I don’t have any money”. The government reimburses extractions, [so] it doesn’t cost them anything.* [CB1].

*Each time a patient needs a root canal and it’s not paid for by social assistance, well, obviously, they never have it done. So then, we have to extract teeth, often even those of children, of adolescents. To remove permanent teeth. That, I would have to say, has been my worst experience*. [CL11].

A perceived lack of motivation in people on social assistance is the other major barrier to the provision of appropriate care. According to our dentists, many patients do not consider oral health as a priority and consequently neglect themselves. Furthermore, despite high treatment needs, they do not consult a dentist often enough and tend to interrupt their episodes of care.

*Most of the time, I find they’re not motivated enough. Their* [oral] *hygiene is very poor. Even though dental services are free, not everyone takes advantage of them.* [CL4].

Dentists consequently feel powerless, emphasizing how challenging it is to perform adequate treatments and, more generally, to improve this clientele’s oral health. These patients, according to them, are not receptive to their recommendations and generally fail to improve oral hygiene and dietary habits. In addition, people on social assistance sometimes ask for treatments, such as multiple extractions, that dentists do not recommend, which is a source of conflict and misunderstanding. In brief, dentists perceive treating people on social assistance as an uphill battle, which makes some conclude that they are wasting their time with these patients.

*When someone comes to you and says,“This tooth hurts, it’s swollen”, you look at the rest of the mouth and, yikes, you wonder where to start; that’s not pleasant. That’s what we see with people on social assistance.* [CL21].

*It’s as if I’m wasting my time talking to a wall.* [CL7].

Feeling constrained to emergency treatments or very basic care, some dentists conclude that there are two types of dentistry: dentistry for the poor and dentistry for everyone else. “Dentistry for the poor” is seen as a devaluation of their competencies and constitutes a source of frustration; not only does it lessen dentists’ satisfaction with their work, but dentists also point out that it has negative impacts on the oral health of people on social assistance.

*So we can’t apply everything we’ve just learnt in school, and therefore we’re forced to practice two kinds of dentistry: dentistry for our regular clientele and dentistry for our welfare clientele. It’s frustrating for the dentist.* [CL9].

### Financial issues - Dentists perceive patients on social assistance as non lucrative and a threat to the financial sustainability of their clinic

Dentists argue that treating people on social assistance is not lucrative because government payment rates—which do not follow the fee guide produced by the dental association—are too low. For some procedures, the fees do not even cover the expenses related to the materials and the staff’s salary.

*I like doing good quality work but, unfortunately, with the fees that social assistance gives us, I mean, we’re talking 12 dollars for an extraction and 5 dollars for the second one, that barely pays for the anesthesia and your muscle power. And that is if it’s an easy extraction. So even for restorations, exams, cleanings, everything is really a lot less. So, if we want to do a good cleaning, then what the government pays is not much.* [CL7].

Most dentists perceive this situation as inherently unfair. They argue that, even though the choice of treatments is limited for people on social assistance, they will not compromise the quality of the procedures they perform, nor can they reduce the cost of the materials and the equipment that are used. Feeling underpaid, some dentists conclude that they subsidize the government.

*That’s what’s frustrating for a dentist. I’ll perform an extraction that, to my mind, is worth the price I would charge you, say, but when I perform the same extraction for a person on social assistance I’ll get paid maybe a fourth of what it’s worth. So it’s frustrating. Of course it’s the person on assistance who suffers the consequences in the sense that we’re maybe frustrated…* [CL2].

Another reason why dentists do not perceive people on social assistance as lucrative patients is related to missed appointments. Missed appointments not only represent “lost wages”, as dentists are not paid for work they cannot perform, but also prevent them from treating “regular patients” who would pay better fees.

*You know that [insurance for those on social assistance] pays about a third of the standard rate of the Dental Association. […] I don’t mind. But when they miss their appointments, then I pay for it three times over. I am already only getting a third as it is and then… I lose a regular patient that could have had that spot, stuff like that.* [AL6].

In brief, dentists perceive people on social assistance as a potential threat to the financial sustainability of dental practices, and some express their relief at having only a small number of such patients. A few, moreover, express concern that this clientele could compromise their financial stability.

*But I can’t of course complain because I don’t have many of them. So if I have one once in a while I don’t mind treating them, but if I had ten a day I’d be stressed out. It would annoy me.* [CL2].

## Discussion

**Figure 1:**
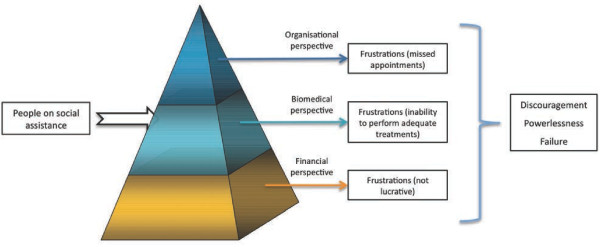
Prism through which participating dentists perceive people on social assistance.

Our study provides an in-depth and unique understanding of dentists’ perspective concerning patients on social assistance: 1) dentists seem to perceive patients through an interpretive filter that distinguishes three important dimensions: organizational, biomedical, and financial (Figure 1); 2) they repeatedly experience frustration and failure along all three dimensions when treating people on social assistance; 3) as a consequence, they tend to feel discouragement and powerlessness, which result in a reluctance to treat people on social assistance, despite their desire to treat all members of society; 4) dentists attribute the difficulties they encounter to the attitude and actions of people on social assistance, whom they perceive as neglecting themselves and disrespecting others, and the health care system, which arguably fails to provide adequate coverage and fees.

With respect to limitations, it is important to note that our study reports the experiences and perspectives of a relatively small number of dentists, even though the size of the sample is adequate considering our methodology [18]. Let us also mention that the sample may not be representative of Canadian dentists. In particular, it did not comprise dentists that systematically refuse to treat people on social assistance, a practice that has not been documented in Canada but has been observed in the United States [6,17] and France [24].

Our team of experienced researchers and highly skilled interviewers employed a series of procedures that enhance credibility [18,25], such as prolonged engagement of the researchers in the community of private dentists, peer-debriefing after the interviews, rigorous data coding, and triangulation of interpretations. Finally, the inductive nature of our approach provided data whose depth could not have been sounded through traditional quantitative research.

Even though studies have already shown that missed appointments, poor compliance, and low reimbursement rates may lead North American dentists to exclude people on social assistance, our research provides results that are original. First, it describes the complexity of dentists’ perceptions in a way that has not been shown. For each of the three dimensions, dentists experience failures that tend to accumulate. These failures also generate emotional reactions among oral health professionals, such as anger and discouragement, which may impact on their relationship with patients on social assistance.

Second, our study highlights the importance of a biomedical dimension: dentists explain how challenging it is to improve the oral health of patients on social assistance. Furthermore, some feel constrained, performing a “dentistry for the poor” that does not reach the standards of “regular dentistry”. Most dentists express their aversion to the former, which is limited to rudimentary and low quality treatments. In particular, because the Quebec public dental insurance program is not comprehensive, dentists must often extract teeth that could be restored if the patients were able to pay. This type of situation creates major ethical dilemmas for professionals trained to achieve high standards of care; it also generates feelings of devaluation.

Third, most dentists perceive themselves as victims with little power to resolve the issues they describe. Instead, they criticize an inadequate health care system; reimbursement rates for people on social assistance are too low and not all dental procedures are covered. They also blame people on social assistance: missed appointments, emergency visits and lack of availability during off-peak hours are considered signs of self-neglect and disrespect for others. For instance, some participants mentioned laziness as a reason for missing appointments, which reflects common stereotypes about poverty [26]. It also echoes the painful sentiment of people on social assistance: they feel misunderstood by oral health professionals [10,13,14,27] and, in a more general way, by society at large [28,29]. In fact, many people on social assistance actually work several hours per week in work reintegration programs, and therefore have limited availability for appointments during the day. This “blaming the victim attitude”, which has already been shown among physicians [30], reflects professionals’ misunderstanding of poverty and exclusion.

In order to increase dentists’ willingness to treat people on social assistance and improve their access to dental care, solutions addressing all three main issues are needed. We must consider and respond to dentists’ complaints, in particular the fact they blame both people on social assistance and the public dental care system for the organizational, biomedical, and financial issues described in this article.

To address the tensions and conflicts between dentists and people on social assistance, we suggest: a) helping current [31] and future professionals – through dental education programs [2] – to better understand people living in poverty and avoid victim blaming [32,33]; b) inviting dentists and people on social assistance to confront their perspectives and together develop solutions that will facilitate their clinical relationships; joint problem identification and solutions might be found by bringing all stakeholders together in what is referred to as participatory action research.

In addition, we suggest addressing current limitations of the health care system by improving coverage and fees. It is worth noting that, in the United States, augmented Medicaid payment rates are associated with dentists’ increased participation in the program as well as increased utilization of dental services by Medicaid recipients [17,34].

Several successful programs in the United States, such as Access to Baby and Child Dentistry (ABCD) [35] and Head Start [27], constitute strong bases leading to better oral health outcomes; modified versions of these programs could be expanded to serve adults as well.

## Conclusions

In conclusion, we would emphasize the common goals uniting our research with the principles upheld by the American Dental Education Association [36], who stated in 2010 that “access to basic oral health care is a human right”; “the oral health care delivery system must serve the common good”; and that “the oral health needs of vulnerable populations have a unique priority”. According to these principles, the current situation is unacceptable, and we urge public health planners, governmental bodies, and community groups to join forces with the dental profession in order to implement concrete solutions. We also invite researchers to contribute to this effort and perhaps even lead such processes. In particular, we suggest the development of participatory action research projects that will allow people on social assistance, clinicians, and policy makers to confront their perspectives and together find solutions.

## Competing interests

The authors declare that they have no competing interests.

## Authors’ contributions

CB originated the study, directed all aspects of its implementation, and led the writing of this article. CL, PA and LR assisted him. CL and AL organized the interviews and the analyses. All authors interpreted findings and reviewed drafts of the article. All authors read and approved the final manuscript.

## Pre-publication history

The pre-publication history for this paper can be accessed here:

http://www.biomedcentral.com/1472-6963/13/464/prepub
